# Impressions of American Surgery

**Published:** 1875-05

**Authors:** John Eric Erichsen

**Affiliations:** Senior Surgeon to the Hospital, and Professor of Clinical Surgery in the College


### Selections.

### IMPRESSIONS OF AMERICAN SURGERY.

An Address delivered at University College Hospital, Nov. 9, 1874,

By JOHN ERIC ERICHSEN, F.R.C.S.,

Senior Surgeon to the Hospital, and Professor of Clinical Surgery in the College.

Gentlemen—I have been requested to give you an account of the impressions that I have formed of American Surgery and Surgical Institutions during my recent visit to the United States; and although I did not go amongst our Transatlantic brethren as "a chiel amang them takin' notes," and still less with any intention to "print 'em," but solely for recreation in my autumn holiday, yet I have no hesitation in complying with this request: partly because I believe that too little is known of American surgery in this country; and partly because the opportunity is thus afforded me, which might not otherwise occur, of publicly expressing my deep sense of the great honor that was conferred on me by the most flattering reception I met with from the medical profession collectively in every part of the Union that I visited. It would be unbecoming here to do more than allude to the many friendly and cordial acts of private hospitality that were extended to me by individual members of our profession, and which have left a deep and pleasing recollection in my mind. But I may, without restraint, express my acknowledgments to the profession in America for the warm and hospitable and hearty welcome that was given to me wherever I went, east or west, north or south, from New York to Chicago, from Boston through Philadelphia and Baltimore to Washington; and for the splendid hospitalities of which I was in most of these cities on several occasions the recipient, as the guest of institutions or of the general body of the profession. I can only explain the reception I thus met with—a reception unparalleled, I believe, in the social history of our profession—on the assumption that it was not to me individually that so much honor was intended to be done, but that a compliment was thus paid to the surgical profession of Great Britain, of which I was considered the representative, and which was thus honored through one of its members by our American brethren. But I must leave these topics, agreeable as it may be for me to dwell upon them, and grateful as the recollections conjured up by allusions to them are, and proceed to tell you, in a few words, what my general impression is of the profession, the schools, and the hospitals of the United States.

And first, as to the Profession, I may at once say that it appears to me to occupy in America, relatively to the rest of the community, a far higher social status than it does in this country. The reason for this seems tolerably obvious. In the absence of an exalted hierarchy in an established church and of great dignitaries of the law, these professions do not offer sufficient inducement for men of the highest intellectual calibre to enter them. Medicine therefore stands prominent as probably the best-educated, certainly the most scientific, and consequently, in a country where education is so widely diffused and so much regarded, the most respected, of the professions. And in the absence of all titled classes, it can socially more than hold its own in competition with the trading and financial elements which are such prominent constituents of the society of most of the American cities. Perhaps, also, the high position that medicine occupies is owing, in some respects, to the greater uniformity of practice that prevails amongst medical men in America than with us. For, just as in the Law there is no division into barristers and solicitors, so in Medicine there is none into physicians, surgeons, and general practitioners. Special aptitude, inclination, or opportunity will necessarily lead men to a greater eminence in particular departments of the profession. But the subdivision into classes and specialties, which is so prevalent here, is unknown in the United States. With the exception of hospitals for diseases of women, I know of no special hospital in any of the large American towns; and I think that no better proof can be adduced of the inutility of the multiplication of these pseudo-charities, which are too often established in order to foster private gain under the flimsy shelter of that much-worn "cloak" which "covers a multitude of sins," than this fact, that in a population larger than that of Great Britain, with a numerous, highly educated, and active profession, it has not yet been found necessary to institute such establishments.

Surgery in the United States certainly stands at a very high level of excellence. The hospital surgeons throughout the country have struck me as being alike practical, progressive, and learned in a very high degree. In practical skill, and aptitude for mechanical appliances of all kinds, they are certainly excelled by no class of practitioners in any country. They are thoroughly up to modern surgery in its most progressive forms, and I have never met with any class of men who are so well read and so perfectly acquainted with all that is done in their profession outside their own country. It would be a great injustice to American surgeons for it to be supposed that surgical skill is confined to the large cities or to the few. On the contrary, I know no country in which, so far as it is possible to judge from contemporary medical literature, there is so widely diffused a high standard of operative skill as in the country districts and more remote provinces of the United States. The bent of the mind of the American surgeon is, like ours, practical rather than scientific; in fact, there are the same mental characteristics displayed in him that we find here—the same self-reliance, the same practical aptitude, the same *curative* instinct, which leads him to consider his patient rather as a human being to be rescued from the effects of disease or injury than as a scientific object to be studied for the advance of professional knowledge. How, indeed, can it be otherwise than that there should be such a resemblance? It is true that in traveling through America one is struck by the fact that there is a singular combination of the new and the old—of the strange and the familiar. That there are differences of a remarkable character between the New and the Old World there can be no doubt. I use the word "different" rather than "foreign," because I feel it impossible to apply the word "foreign" to anything American. There are differences in climate, differences in the physical configuration of the country. The verdure that clothes its hills and the vegetation that fertilizes its plains are different from those that we meet with here; but man, in all his characteristics, is exactly the same. There appears to me, indeed, to be as great, if not a greater difference between the mental characteristics of an Englishman and some of the other inhabitants of Great Britain than there is between an ordinary Englishman and an American of the Atlantic cities. It may be truly said, though perhaps in a sense slightly different from that in which the poet used the words, that

"Cœlum non animum mutant qui trans mare currunt."

Those who have crossed the great ocean have changed their clime, but not their characters.

The similarity that exists between American and British surgery, and which has struck me very forcibly, arises not only from the great resemblance that exists between the American and the English character, but from two other causes which have largely contributed to this end. The Art of Surgery is in a great measure traditional. The method of doing things in surgery is transmitted directly from the master to the pupil. The American surgeon of a past generation acquired in this way the traditionary art of British surgery, and has transmitted it directly to his descendants. Surgeons of both nations drew their inspiration from the same source and drank at the same fountain of knowledge. The names of Cooper and the Bells, of Liston and of Brodie, are as familiar to the ears of American surgeons as they are to those of this country. I was much struck when visiting the oldest hospital in the United States—the Pennsylvania General Hospital at Philadelphia—by seeing over the entrance to the operating theatre the portrait of a face that I had often seen delineated in this country. At first I thought it must be that of one of the American surgical worthies of a past generation—of Physick or of Mott, of Warren or of Mutter; but on closer inspection I found that they were the well-known features of him who was in his generation *facile princeps* of British surgery—Sir Astley Cooper. Not only have British traditions thus penetrated deeply into the surgery of the United States, but the modern American surgeon derives his information from the same sources as does his British contemporary. *The Lancet* is reprinted, and is as widely circulated in the States as in this country; and I find in my own case that my pupils in America are probably more numerous than those in Great Britain. One of the great advantages—and it is a very great one—that an English writer enjoys, is that he addresses eighty millions of people, and that his works are not only disseminated throughout his own country, but, if of any value or importance, are eagerly sought after by that still larger body of readers existing in the "Greater Britain" which now encircles the globe. And if it be true, as has been said, that the judgment of enlightened foreign contemporaries is an anticipation of that which posterity will give, he may posibly have a foreshadowing of the verdict that a future generation of his own countrymen will pass upon him, in the estimate in which he is now held amongst those who inhabit the regions beyond the Atlantic.

The mode of instruction in the Medical Schools of America is necessarily very much the same as here. But there are some differences. Thus, the course of education required is generally shorter than with us, and no preliminary examination in the way of matriculation is required before a young man can enter upon his medical studies. This undoubtedly is a great evil, and as such is much deplored by many teachers to whom I have spoken on the subject; but I am told that, in the present state of things in America, it would be impossible to sift the candidates for a profession, or to establish such an ordeal as a matriculation examination. About the systematic instruction I have nothing to say. There is no essential difference in this respect between England and America. It is everywhere much the same. Dissection is easy to be obtained, and the bodies used for that purpose are either furnished gratuitously to the students or supplied at a low cost. There is but a nominal fee required for hospital attendance. The whole system of medical education, indeed, is far cheaper than with us. The chief difference that I have observed is in the method of communicating clinical instruction. The medical schools in the principal cities of the States, especially in New York and Philadelphia, are so enormous that the classes would be too large to be conducted through the wards of a hospital. Classes numbering from 600 to 800 are not uncommon. At Professor Pancoast's introductory lecture at Jefferson College, Philadelphia, there were probably 600 students present; and at my last visit to the clinical theatre at Bellevue Hospital, New York, it was estimated that a thousand studen's were present. It therefore becomes necessary to brin .he patient to the students, rather, than, as with us, the pupils to the patient. This is done by raising him off the framework of his bed on a small platform running on wheels, and thus carrying him without any disturbance of position into the clinical theatre, where he is examined and the case discussed. I will give no opinion as to the value of this method of teaching in medical cases, but in many surgical cases it appears to me to be far more useful than that which is generally adopted in this country. The surgical disease or injury can be more readily displayed in this way to a large body of students than it can when they are crowding round a bed, standing in one another's way, and often hampering the proceedings of the surgeon. There is one peculiarity in the mode of instruction in American hospitals which deserves mention. It is this: that each surgeon of the hospital often only serves for a short period of the year—four to at most six months; each of his colleagues in rotation taking up the practice and the clinical teaching during the remainder of the term. The advantage of this system is, that the teacher is not exhausted by continuous work, but returns with refreshed energy to his duties.

The Hospitals in the United States are, as with us, supported by voluntary contributions or by endowments from wealthy benefactors. The Americans are munificent in their charity, and hence these institutions are numerous and well organized. America has two sets of hospitals, the old and the new. Like England in some of its larger towns, it is still embarrassed by the hospitals erected in pre-sanitary days, under systems of construction which time, experience, and the advance of scientific knowledge have proved to be erroneous, in which septic diseases are readily generated and become largely destructive to the patients. These institutions are, however, undergoing a process of conversion which will speedily do away with many of the evils inseparably connected with such buildings. The Americans learned a hard lesson in the deadly struggle of the War of Secession—a lesson which is not likely soon, if ever, to be forgotten by so practical a people, unfettered by old prejudices and preconceived opinions. The lesson to which I allude was this: that wounded and injured soldiers could only safely be treated in the open, in hut or barrack hospitals. This lesson has been taught to Europe by the more recent experiences of the Franco-German war, with what results in the future yet remains to be seen. That the barrack or hut system is superior to any other for surgical cases there can be no question. The misfortune is, that in large towns, where ground is expensive and difficult to be procured, this system is, owing to the space required, not easy of adoption. There are also certain obvious inconveniences connected with it in those cases in which clinical instruction is required to be carried on in the hospital. The problem, then, to be solved in the construction of modern hospitals in large cities appears to be, how to combine the best condition for the patient with economy of space and proper facilities for the student. This problem is in process of solution in America. Indeed, it has, I believe, already been satisfactorily solved.

The new hospitals in the United States are generally built upon a uniform plan, having a central building for the administrative department, and wings for the reception of patients. This is the general plan of the construction of such hospitals as the Presbyterian, Mount Sinai, St. Luke's, and others at New York; of the Episcopalian and the New University Hospital at Philadelphia,—and is in accordance with the best principles of hospital construction as practiced in Europe. But these hospitals, magnificent in construction and in all their appliances as some of them are, have not been found sufficient to satisfy the sanitary requirements of American surgeons. Accordingly the barrack system for surgical cases is now being adopted. It is proposed to erect, in the gardens of the Bellevue Hospital at New York, a detached barrack building for the reception of cases of surgical operation and injury. And a similar detached ward will be constructed in connection with the Hospital for Women, where, under the energetic action of Marion Sims and of Emmett, the number of operations has increased very greatly of late years. At the Presbyterian Hospital in Philadelphia such a building is in course of erection, and will be completed by the commencement of next year. At the Roosevelt Hospital, New York, without exception the most complete medical charity in every respect that I have ever seen—a hospital the construction of which reflects the greatest credit upon its designers,—in this hospital, which is constructed on the plan of a central administrative department, with lateral pavilions, there is a large detached barrack-ward erected in the garden, having no communication with the general structure except through an open corridor. This barrack building or ward is devoted solely to the reception of acute surgical cases. It consists mainly of one large ward, containing thirty-six beds, arranged two and two on either side in the interspaces between the windows. It has an open basement, and a large ventilating space between the ceiling and the roof; and every appliance that modern science can suggest, in the way of securing efficient ventilation, cleanliness and warmth, has been expended upon it. This ward, filled with surgical cases, has now been opened for nearly three years; and I was told by Dr. Weir, who kindly took me over it, that during that time there had only been one case which was supposed to be pyæmic—a case of so-called "pyæmic meningitis" following urethrotomy; any way, a case of blood-poisoning probably rather from self-infection than from external contamination. In addition to this magnificent barrack-ward there is in the gardens attached to the Roosevelt Hospital a separate hut for the reception of erysipelas cases that may be brought to, or might accidentally develop in, the institution. The Roosevelt Hospital appears to me to be a model that might with great advantage be copied in this country, especially in those towns where it is becoming necessary to destroy pyæmia-infected infirmaries and to construct new hospitals. It is a perfect model for a hospital of from 150 to 250 beds ; and the plan admits of its indefinite augmentation by the addition of pavilion wings and barrack-wards.

But admirable as this institution is, an attempt has already been made to improve upon it. The American surgeon, as I have already told you, is progressive. He invariably tries to go from good to better. He will not remain content with those appliances and means which might have been sufficient for a past generation, but are no longer equal to the requirements of the present day. Dr. Billings, of the United States army, one of the most learned and advanced surgeons of the day, is superintending the construction of a hospital at the "Soldiers' Home" near Washington, a magnificent establishment, the Chelsea of America. There is no barrack or hut attached to this hospital, as it is intended for chronic cases only ; but, should occasion require, such a building might easily and cheaply be run up in connection with it. I will mention to you some of the principal points that Dr. Billings is carrying out in connection with this hospital, as they are, I believe, quite novel in this country, although some of them have been adopted in the more recently constructed institutions in the United States, which I have already mentioned. The points are these :— The hospital, like all modern American ones, has a central administrative building and two wings or pavilions for the wards. It is three stories high. The central administrative building contains the staircase, lifts, furnace-chimney and air-shafts. The basement story throughout the building is open ; and the remarkable point in the construction of the hospital is this, that all the domestic offices, such as the kitchens, laundries, etc., are at the top of the building, under the roof on the third story. In this way there is no possibility of contamination of the atmosphere of the hospital from these domestic sources. The first and second stories of the pavilions are occupied by the wards. The walls are parianized; no angles are left in them to harbor dust and dead air, but they are carefully smoothed and rounded off. The ventilation of each ward is independent, and separated from that of the main centre building, and of every other ward. The waterclosets and lavatories, which will be of the most modern construction, also possess an independent system of ventilation. The system of ventilation is briefly as follows:—There is a large brick air-shaft about six feet square, running up the centre of the administrative building, through the middle of which an iron flue ascends, carrying up the smoke and the heat from the furnace below, which is used for heating water that is required for warming the house and for the working of a small steam engine employed for the general purposes of the establishment. The air in the brick shaft is thus always kept warm, and an upward current is established. Into this main shaft, lateral air-shafts, about two feet square, run from each ward. They lie between the floor and the ceiling, communicating with the ward by traps, which can be opened or shut at pleasure, and so arranged that the air can be drawn from the top or bottom of the wards as may be required. Fresh air is pumped into the wards through a large air shaft opening 200 feet from the hospital, into which the air is driven by a fan worked by a small steam engine, 5,000 cubic feet per bed per hour being thus thrown in and passed through the wards. In winter the air can be warmed by passing over coiled hot-water tubes; in summer it can be cooled by being iced. The isolation of each ward is complete. It is effected by means of a transverse corridor stretching across the whole depth of the building between the ward and the central compartment, open at both ends, and shut off from the ward by double swing doors.

In all the modern hospitals that I have visited in the United States, I have been particularly struck with the extreme attention to cleanliness in all that concerns baths, lavatories, and waterclosets. These appliances are as perfect as they are substantial, and as ornate as are to be found in the best private houses. They are invariably carefully isolated from the wards. Great attention is also bestowed upon the disposal of foul linen, which, as a rule, instead of being carried through the building, is conveyed directly from the ward down a separate lift in the central department. The laundries also are models of cleanliness, and generally detached from the main building. The whole of the washing of the clothes, drying, etc., is rapidly done by steam machinery. Hot air chambers are also provided for the rapid drying of damp ward linen. The floors are of hard wood, and are dry-rubbed. Fire-proof staircases and hydrants having a continuous water supply are ready in case of emergency. In many hospitals there are spacious wooden piazzas or verandahs running round the building, and communicating directly with the wards, for air and exercise in summer.

There is a peculiar system adopted in many of the modern American hospitals—having private rooms for the reception of patients who can afford to pay. The charge for these rooms varies from two to five dollars per day. The patient is under the care of one of the medical officers, who receives his fees as he would in an ordinary private attendance. The system is well suited, in cases of sudden emergency, to a country in which there is so large a floating population as in the States, but it is also adopted in cases of chronic disease, as a matter of convenience by many who come from a distance for operation or treatment.

Surgical practice in America does not differ in any very essential respects from that adopted here. There are necessarily some modifications, and many ingenious appliances; but essentially there is no greater difference between American and English surgery generally than is to be found between the practice adopted in any two London hospitals.

The treatment of wounds is sufficiently simple, and presents nothing peculiar. I observe that American surgeons are careful about the drainage of wounds, and employ drainage tubes or similar appliances freely.

"Antiseptics" do not appear to be much, if at all, employed; at least, in a methodical form. Carbolic acid, in the form of lotion or wash, is commonly used. Indeed, antiseptics are not so much needed in the American hospitals as in ours. The object of antiseptics is to prevent the contamination of a wound by septic impurities from without. These sources of contamination do not exist in such hospitals as those I have been describing to the same extent that they do in less perfectly constructed and less hygienically conducted establishments, and hence antiseptics are proportionately less needed. In America it is attempted to accomplish by improved construction of hospitals, and by close attention to hygenic requirements, those great results which we are here driven to attain by "antiseptic" methods of treatment. In consequence of the ignorance in all matters that relate to the hygiene of hospitals that prevails among the architects and managers of these institutions, an undue burden of anxiety, responsibility and care is thrown upon the surgeon, who is now unceasingly engaged in combating septic disease ; and in order to keep down that rate of mortality which is the direct consequence of septic hospital influences, he is driven to the employment of elaborate and complicated methods of antiseptic treatment. Cleanliness in its broadest sense is the best and most efficient antiseptic. If the constructors and conductors of hospitals were acquainted with or would adopt those hygienic rules on which hospitals should be built and managed ; if hospitals were not overcrowded ; if the system of ventilation was perfect; if there was a continuous water-supply, and a proper isolation of wards and distribution of patients, the causes of septic diseases would not be generated. Those foul and filth-begotten diseases, pyæmia and hospital gangrene, would disappear, and antiseptics, in the absence of septic influences, would become unnecessary. Contamination of hospital air would be prevented ; we should not, as now, under defective hygienic arrangements, first allow the pollution to take place, and then be driven to the use of antiseptics in order to prevent infection of wounds by the already septic-laden atmosphere. Under the present system we begin at the wrong end. Instead of preventing the possibility of atmospheric contamination by perfect hospital hygiene, we allow the septic poison to be engendered, and then, before it can be implanted on the wound, seek to destroy it by the employment of chemical agents.

With regard to Anæsthetics I have little to say. Ether is invariably used at Boston, and is preferred to chloroform by many hospital surgeons in other American cities. I saw it given in some operation cases at the Massachusetts General Hospital, which has the great honor and privilege of being the institution in which anæsthesia was first employed in any surgical operation ; it was administered here on a sponge, without any apparatus or complicated contrivance.

Except in Boston the Museums are still in an undeveloped condition. That of Dr. Mott, at New York, was unfortunately destroyed by fire; but Dr. Wood, with characteristic energy, is trying to establish one at Bellevue. At Philadelphia the splendid collection of Hyrtl's injections have lately been acquired by purchase. But there is one museum which is so unique, so admirably arranged, and so interesting, that I must direct your attention to it for a few minutes. It is the Museum of the Army Medical Department at Washington. This magnificent collection, illustrating not only every possible variety of gunshot and arrow injury, but also those diseases which are more fatal than the bullet to an army in the field or camp, has, under the able superintendence of Surgeon-General Barnes and of Drs. Otis and Woodward, been most admirably arranged and catalogued. It occupies a building that has a melancholy interest connected with it, as being the theatre in which President Lincoln was assassinated by Booth in 1865. The collection itself is well known in Europe through the medium of those beautifully illustrated and ably collated medical histories of the great War of the Rebellion which have been published under the superintendence of the Medical Department of the United States Army. Many of the specimens in this museum are quite unique. I would especially refer to a series illustrating splintering of the inner table of the cranium without fracture of the external table consequent on contusion of the skull; the splitting of conical bullets against sharp edges of bone; a collection of foreign bodies, including arrowheads, forming the nuclei of calculi extracted from the bladder; and a remarkable series of specimens of injuries of the bones inflicted by arrow-wounds in Indian warfare. To the physician the collection of diseases of the large and small intestine, resulting from dysentery, diarrhœa, camp fevers, typho-malaria, etc., is most interesting. The preparations have been admirably put up by Dr. Woodward. In this collection will be found some very interesting specimens of resection of joints and bones after gunshot injury. I saw one patient in whom the head of the humerus and several inches of the shaft—in all, seven inches of the bone—had been excised. He was now a porter in the museum, had a most useful and well-developed forearm and hand, serviceable for every purpose. There was not only the preparation, but the living example of this triumph of conservative surgery. The case illustrates the important fact that the upper end of the humerus may be resected below the insertion of the deltoid and yet a useful limb be left. I saw another case of an officer who had lost about four inches of the shaft of the humerus, and in whom the limb was equally useful. In fact, American surgeons are very skilful in the management of resections. Sayre has removed the head of the femur fifty-two times, and in his wards I saw some admirable results of the operation, due in a great measure to the very careful after-treatment to which he subjects the patient, and on which he lays great stress.

I have thus given you a very brief sketch of some of the impressions that I formed of our profession during my recent visit to America, and in so doing I have purposely, as far as possible, omitted mentioning the names of American surgeons, because I felt that there are so many so highly distinguished that it would be invidious and perhaps unjust to make a selection of a few amongst the juniors, and amongst the seniors it would be needless to name to you such men as that Nestor of American surgery, Gross, or of Pancoast, of Philadelphia; of Van Buren, Wood, Parker, or Sayre, of New York; Bigelow or Hodges, of Boston; Smith or Johnston, of Baltimore. I can only say that the surgical profession in America contains a phalanx of men alike distinguished for their skill and their knowledge, at least equaling what any European country can produce. And, in conclusion, I would advise those amongst you who wish to see and study the practice of surgery elsewhere than in the school in which you have been brought up in this country, who are not content throughout their lives *jurare in verba magistri*, to run in the one professional groove in which they have been launched, but who unfortunately have not acquired that fluency of the speech of Germany or of France that would render a residence in those countries profitable for the purposes of study, to take a trip across the Atlantic—a voyage in itself interesting, amusing, and health-giving,—and to spend a few months in visiting the great hospitals and schools in the cities of the United States of America.—*London Lancet.*

